# Combinatorial Efficiency Evaluation: The Knapsack Problem in Data Envelopment Analysis

**DOI:** 10.1155/2014/789053

**Published:** 2014-07-09

**Authors:** Xiao-guang Qi, Bo Guo

**Affiliations:** College of Information System and Management, National University of Defense Technology, Changsha 410073, China

## Abstract

The traditional data envelopment analysis (DEA) literatures generally concentrated on the efficiency evaluation of single decision making unit (DMU). However, in many practical problems, the decision makers are required to choose a number of DMUs instead of a single one from the DMUs set. Therefore, it is necessary to study the combinatorial efficiency evaluation problem which can be illustrated as a knapsack problem naturally. It is indicated that the basic model proposed by Cook and Green may have some drawbacks and a modified model, which is combined with the super efficiency model, is proposed in this paper. What is more, our proposed model is more persuasive to the decision makers because it is able to provide a unique best combination of DMUs. An adapted local search algorithm is developed as a solver of this problem. Finally, numerical examples are provided to examine the validity of our proposed model and the adapted local search algorithm.

## 1. Introduction

Data envelopment analysis (DEA) was first introduced by Charnes et al. [1] in 1978. DEA is an effective method of evaluating the relative efficiency of decision making units (DMUs) which consume multiple inputs to produce multiple outputs. After more than thirty years' development, DEA has become a significant and active research area; see [2–4] as reviews of DEA. The traditional DEA literatures, which we called individual efficiency evaluation here, generally concentrated on the efficiency evaluation of single DMU. However, in many practical problems, such as projects selection or technology evaluation problem, the decision makers are required to select a number of DMUs instead of a single one. According to Cook and Green's research [5], traditional individual efficiency evaluation is not adequate to support the decision making in these problems mainly because a combination of individually efficient DMUs is not necessarily still efficient within all the possible combinations. Therefore, it is necessary to do further research on the combinatorial efficiency evaluation problem which means the efficiency evaluation of multiple DMUs combined together under the DEA framework and can be illustrated as a 0-1 knapsack problem naturally [5].

The combinatorial efficiency evaluation is a relatively new problem and some related researches can be found in the projects selection and technology evaluation problem. Oral et al. [6] firstly proposed a DEA-based multistage methodology in the R&D projects collective evaluation and selection problem. Cook and Green's [5] research afterwards may be the primal article that combined the DEA model and knapsack problem together to solve the R&D projects selection problem. Loch et al. [7] and Beaujon et al. [8] did their researches on the projects selection problem with different mathematical programming models. A DEA-based methodology was developed by Eilat et al. [9] considering the interactions between R&D projects. Vitner et al. [10] used DEA to compare the project efficiency in a multiproject environment. Based on Cook and Green's research, Chang and Lee [11] extended the problem into the fuzzy case. DEA was also used in a methodology proposed by Khalili-Damghani et al. [12]. Tavana et al. [13] introduced a fuzzy DEA model for a high-technology projects selection problem at NASA.

Cook and Green' paper [5] is considered as a basic research on the combinatorial efficiency evaluation problem; some other papers [11, 13] are based on their research. However, during our research, we find that there may be some mistakes in Cook and Green's model. As mentioned in [10], each project in the projects selection problem is necessarily a one-time nonrepeated event. But in Cook and Green's model, this basic fact was neglected and the same project was possible to be selected more than once. Considering the drawbacks of Cook and Green's model, we proposed a new DEA-based methodology which is combined with the knapsack problem model and super efficiency model. Comparing with the previous researches, the efficiency evaluation based on our proposed methodology is more practically based on the fact that each DMU is allowed to appear only once in a combination. Our proposed methodology is also more persuasive to the decision makers because it is able to provide a unique best combination of DMUs. What is more, our proposed methodology is combined with the super efficiency model and therefore is more powerful in discriminating the efficient combinations of DMUs. An adapted local search algorithm is provided as a solver of the knapsack problem.

The rest of this paper is organized as follows: Section 2 is the problem formulation with some comments on Cook and Green's model.  Section 3 illustrates our proposed model in detail.  Section 4 described the adapted local search algorithm briefly.  Section 5 is the application into numerical examples. Finally, we give the conclusions in Section 6.

## 2. Problem Formulation

### 2.1. DEA and Its Extension to Combinatorial Efficiency Evaluation

DEA was first introduced by Charnes et al. in 1978 [1]. It is supposed that, in a DEA problem, there are *n* DMUs with *m* inputs and *s* outputs. The vectors *x*_*j*_ = [*x*_1*j*_, *x*_2*j*_,…,*x*_*mj*_]^T^ and *y*_*j*_ = [*y*_1*j*_, *y*_2*j*_,…,*y*_*sj*_]^T^ are usually used to denote the inputs and outputs of DMU_*j*_, in which *j* = 1,2,…, *n*. And the basic efficiency evaluation model of DMU_*j*_0__  (*j*_0_ = 1,2,…, *n*) is as follows:

(1) Max⁡hj0=uj0Τyj0s.t.uj0Τyj−vj0Τxj≤0, j=1,2,…,nvj0Τxj0=1uj0≥0vj0≥0,

where *u*_*j*_0__ = [*u*_1*j*_0__, *u*_2*j*_0__,…,*u*_*sj*_0__]^T^ and *v*_*j*_0__ = [*v*_1*j*_0__, *v*_2*j*_0__,…,*v*_*mj*_0__]^T^ are the optimal weights assigned to the inputs and outputs, respectively, and *h*_*j*_0__ is the efficiency score of DMU_*j*_0__  (*j*_0_ = 1,2,…, *n*). Although many different forms of DEA models have been developed [2–4], we use the basic CCR model (model (1)) here to illustrate our thoughts on combinatorial efficiency evaluation. Some further studies are possible to extend the combinatorial efficiency evaluation into other DEA models.

It can be found that, just like the basic CCR model, most of the traditional researches are concerned with the individual efficiency evaluation of single DMU but pay no attention to the combinatorial efficiency evaluation of multiple DMUs. As mentioned before, sometimes the decision makers are required to choose a number of DMUs from the alternatives instead of a single one, and, therefore, the combinatorial efficiency evaluation is needed. Before extending our research to the combinatorial efficiency evaluation problem, we give some definitions first for convenience.

**Definition 1 .**

The combinatorial efficiency evaluation (CEE) means the efficiency evaluation of a combination of multiple DMUs under the DEA framework.

**Definition 2 .**

The combinatorial-DMU (CDMU) is a subset of DMUs set and can be seen as a new individual DMU in CEE problem.

**Definition 3 .**

The possible-combination set (*P*) is the power set of DMUs set (excluding the empty set *∅*) which contains all the possible combinations of DMUs, and it is described as follows:

(2) $$\begin{array}{c} P=\prod \left({\text{DMU}}_{s}\right)=\prod \left(\left\{{\text{DMU}}_{1},{\text{DMU}}_{2},\ldots ,{\text{DMU}}_{n}\right\}\right). \end{array}$$

The number of CDMUs in *P* is denoted by |*P*|, and, for CDMU_*k*_  (*k* = 1,2,…, |*P*|), a binary 0-1 vector *c*_*k*_ = [*c*_1*k*_, *c*_2*k*_,…,*c*_*nk*_]^T^ is used to denote the combination as follows:

(3) cjk={1,DMUj∈CDMUk,0,DMUj∉CDMUk, j=1,2,…,n.

It is supposed that all the DMUs in DEA problem are neither synergistic nor interfering [5]; therefore the inputs and outputs of the CDMUs are directly the sum of its elements' inputs and outputs. For example, the inputs *X*_*k*_ = [*X*_1*k*_, *X*_2*k*_,…,*X*_*mk*_]^T^ and outputs *Y*_*k*_ = [*Y*_1*k*_, *Y*_2*k*_,…,*Y*_*sk*_]^T^ of CDMU_*k*_  (*k* = 1,2,…, |*P*|) can be calculated as follows:

(4) $$\begin{array}{c} X_{ak}=\overset{n}{\underset{j=1}{\sum }}c_{jk}x_{aj},\;a=1,2,\ldots ,m\\ Y_{bk}=\overset{n}{\underset{j=1}{\sum }}c_{jk}y_{bj},\;b=1,2,\ldots ,s. \end{array}$$

There are usually some constraints on the inputs and outputs of CDMUs and therefore not all the CDMUs in the possible-combination set *P* are rational in practice. In a CEE problem, what we are concerned with is the CDMUs that satisfied the constraints instead of the entire possible-combination set. For this reason, we give some further definitions about the possible-combination set *P*.

**Definition 4 .**

The rational-combination set (*R*) is a subset of *P* in which the CDMUs satisfied some constraints on inputs and outputs, and it is described as follows:

(5) $$\begin{array}{c} R=\left\{{\text{CDMU}}_{k}\;|\;X_{k}\leq X_{0},Y_{k}\geq Y_{0},{\text{CDMU}}_{k}\in P\right\}, \end{array}$$

where *X*_0_ and *Y*_0_ are the constraints on inputs and outputs of CDMUs, respectively. In some simplified situation, there may be no constrains on the outputs and the rational-combination set can be described as follows:

(6) $$\begin{array}{c} R=\left\{{\text{CDMU}}_{k}\;|\;X_{k}\leq X_{0},{\text{CDMU}}_{k}\in P\right\}. \end{array}$$

**Definition 5 .**

The desired-combination set (*D*) is a subset of *R* in which the CDMUs are not allowed to combine with any other DMU under the restrictions of *X*_0_, and it is described as follows:

(7) D={CDMUk ∣ Xk≤X0,CDMUk∈P, ∀DMUj∉CDMUk,Xk+xj>X0,j=1,2,…,n}.

What the decision makers are concerned with the most is the efficiency evaluation of CDMUs in the desired-combination set *D* that make full use of the budgets. It should be noted here that the DMUs in a CDMU are naturally one-time nonrepeated events and a DMU is not allowed to appear repeatedly in a combination. Therefore, the basic combinatorial efficiency evaluation model of CDMU_*k*_0__  (*k*_0_ = 1,2,…, |*D*|) in desired-combination set *D* is as follows:

(8) Max⁡Hk0=uk0ΤYk0s.t.uk0ΤYk−vk0ΤXk≤0, k=1,2,…,|D|  (8.1)vk0ΤXk0=1uk0≥0vk0≥0.

As CDMU is a combination of DMUs, the CEE model of CDMU_*k*_0__  (*k*_0_ = 1,2,…, |*D*|) can be illustrated as an equivalent 0-1 knapsack problem model as follows, in which the binary 0-1 vector *c*_*k*_0__ = [*c*_1*k*_0__, *c*_2*k*_0__,…,*c*_*nk*_0__]^T^ is the solution we need:

(9) Max⁡ Hk0=uk0Τ(∑j=1ncjk0yj)s.t. uk0Τ(∑j=1ncjkyj)−vk0Τ(∑j=1ncjkxj)≤0,k=1,2,…,|D|vk0Τ(∑j=1ncjk0xj)=1uk0≥0vk0≥0cjk∈{0,1}, j=1,2,…,n,  k=1,2,…,|D|.

There are mainly two drawbacks in model (8). Firstly, as mentioned in [5], the quantity of restrictions (8.1) would grow too fast as the number of DMUs becomes bigger, and this would affect the practicability seriously. Secondly, there would be more than one CDMU to be evaluated as efficient based on the basic model (8). This would be less persuasive to the decision makers to make a choice between the CDMUs. These two problems would also exist in model (9) and some further discussion is needed to improve the usability of models (8) and (9).

### 2.2. Some Comments on Cook and Green's Model

Cook and Green introduced a resource-constrained DEA approach combined with the knapsack problem in the project prioritization problem in order to select a subset of projects from a larger set of proposals [5]. Cook and Green's paper may be the first research about the CEE problem, and some other papers have done research based on Cook and Green's work [11, 13]. However, during our research, we found that there are some drawbacks in Cook and Green's model and some comments are provided in this section.

In a project selection problem, the decision makers are required to select a number of projects with constrains on the project cost. The alternative projects are considered as the DMUs in DEA problem and the individual efficiency of each project can be calculated by model (1). Model (8) was also used as the basic model in Cook and Green's paper and, in order to overcome the first drawback of model (8), Cook and Green introduced the following model:

(10) Max⁡Hk0=uk0ΤYk0s.t.uk0Τyj−vk0Τxj≤0, j=1,2,…,n  (10.1)vk0ΤXk0=1uk0≥0vk0≥0.

Comparing Cook and Green's model (10) with the basic model (8), we can find that restrictions (8.1) were replaced by restrictions (10.1) which are the same with the basic CCR model (1). It is effective to reduce the quantity of restrictions in model (8) but some problems arose too.

The inequality restrictions in a DEA model shaped the efficient frontier of the production possibility set (PPS) which is used as the benchmark of evaluating all DMUs. A fundamental fact, which was neglected during Cook and Green's transformation, is that the efficient frontier has changed during the combination of DMUs. In Cook and Green's model, the combinatorial efficiency of CDMUs is evaluated by the original efficient frontier of DMUs, and this would be inappropriate for some CDMUs. Cook and Green's model also neglected the fact that a DMU is not allowed to appear repeatedly in CDMUs.

In a word, the major drawback of Cook and Green's model is that it had not realized the differences between the PPS of set *R* and set *D*. The purpose of Cook and Green is to evaluate the efficiency of CDMUs in set *D*, but what they used was the PPS of set *R*. It is obviously inappropriate and that will result in some efficient CDMUs being evaluated to be inefficient by error.

In the following, a simple numerical example is provided to illustrate the drawbacks of Cook and Green's model. Suppose that there are 4 DMUs shown in Table 1 with one input and two outputs. The cost of the CDMUs is restricted to be no more than 2 units; therefore, all the possible combinations are provided in Table 2. The purpose of this CEE problem is to evaluate the combinatorial efficiency of CDMUs in set *D*. And the evaluation results by models (8) and (10) are compared in Table 3.

**Figure 1:**
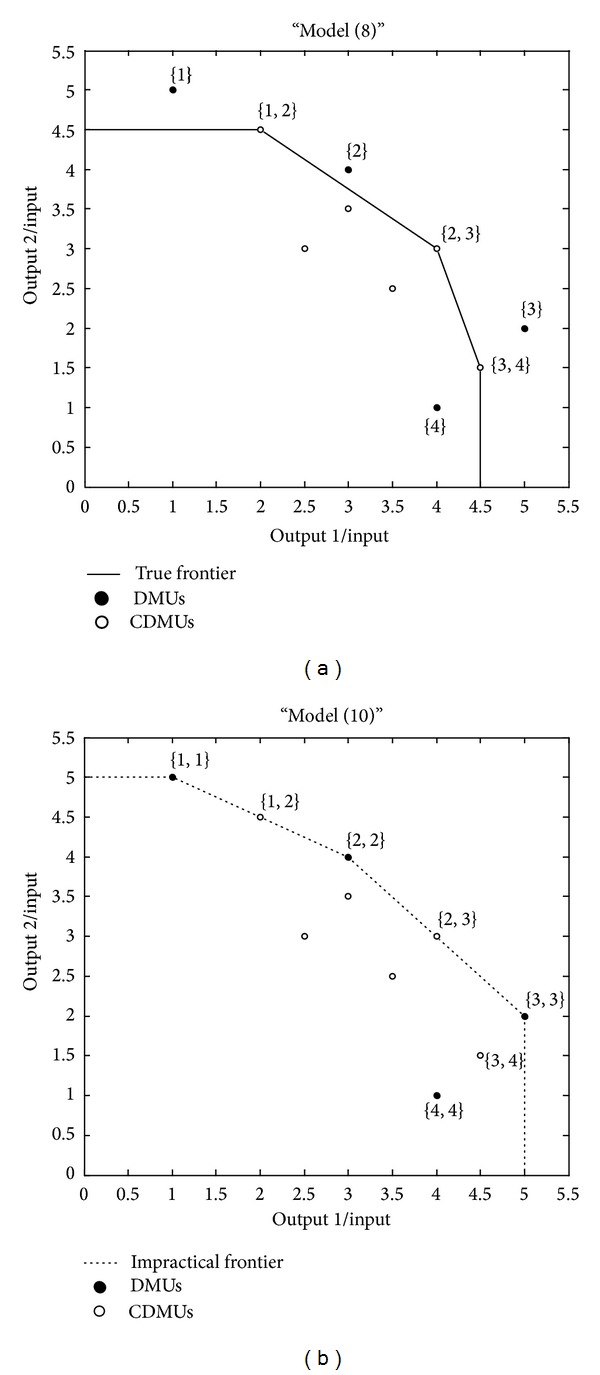
Different efficient frontier in model (8) and model (10).

Comparing the evaluation results in Table 3, we can find that the CDMU {3,4}, which should be efficient in the combination set *D*, is evaluated incorrectly by model (10). This is mainly because some impractical combinations, such as {1,1}, {2,2}, {3,3}, and {4,4}, are used to shape an impractical efficient frontier in Cook and Green's model (10); see Figure 1. And the combinatorial efficiency evaluated by the impractical efficient frontier is certainly incorrect.

## 3. Proposed Model for the CEE Problem

In order to overcome the drawbacks of model (8) and model (10), a new combinatorial efficiency evaluation model is proposed in our paper. There are mainly three advantages of our proposed model: (a) our model is combined with the super efficiency model and therefore the best CDMU can be provided to the decision makers; (b) in the meanwhile, the quantity of restrictions is reduced to be acceptable in our model; (c) finally, evaluation in our model is based on the super efficient frontier and no efficient CDMU would be incorrectly evaluated.

The super efficiency model is a series of DEA models in order to achieve a full ranking of both efficient and inefficient DMUs [14–18]. A basic super efficiency model is provided by Andersen and Petersen in [14] as follows:

(11) Max⁡hj0=uj0Τyj0s.t.uj0Τyj−vj0Τxj≤0, j=1,2,…,n,  j≠j0vj0Τxj0=1uj0≥0vj0≥0.

The main idea of the AP model is to evaluate the efficiency of a target DMU by excluding it from the DMUs set. The practical meaning of AP efficiency is a measure of how much a DMU can extend the PPS. This is persuasive enough to the decision makers in reality. Although infeasibility would happen in some cases and many papers have done research to solve the infeasibility problem [15–18], our interest is not to improve the super efficiency model. It should be noted here that any improved super efficiency model can be used in the CEE problem, and the emphasis of our paper is to provide a methodology of solving the CEE problem combined with the super efficiency model. For this reason, we give our proposed model for the CEE problem as follows:

(12) Max⁡Hk0=uk0ΤYk0s.t.uk0Τyj−vk0Τxj≤0,   j=1,2,…,n, DMUj∉CDMUk0vk0ΤXk0=1uk0≥0vk0≥0.

What is more, the equivalent knapsack problem model can be formulated as follows:

(13) $$\begin{array}{c} \begin{array}{cc} \mathrm{Max} & H_{k_{0}}={u_{k_{0}}}^{Τ}\left(\overset{n}{\underset{j=1}{\sum }}c_{jk_{0}}y_{j}\right)\\ \text{s}.\text{t}. & \left(1-c_{jk_{0}}\right)\left({u_{k_{0}}}^{Τ}y_{j}-{v_{k_{0}}}^{Τ}x_{j}\right)\leq 0,\;j=1,2,\ldots ,n\\ & {v_{k_{0}}}^{Τ}\left(\overset{n}{\underset{j=1}{\sum }}c_{jk_{0}}x_{j}\right)=1\\ & u_{k_{0}}\geq 0\\ & v_{k_{0}}\geq 0\\ & c_{jk_{0}}\in \left\{0,1\right\},\;j=1,2,\ldots ,n. \end{array} \end{array}$$

Model (13) is used as the fitness measure of solutions in the knapsack problem, and the optimal solution *c*_*k*_0__ = [*c*_1*k*_0__, *c*_2*k*_0__,…,*c*_*nk*_0__]^T^ is the result we need. And it is assumed that all the CDMUs we considered here belong to the desired-combination set *D*.

## 4. An Adapted Local Search Algorithm

An adapted local search algorithm is developed in this section to solve the CEE problem with the knapsack problem formulation. The local search algorithm is simple but effective in solving combinatorial optimization problems [19–21]. Considering the fact that the emphasis of our research is proposing a methodology for the CEE problem, the basic local search algorithm is used here with some adaptations. The pseudocode of the basic local search algorithm is provided in Pseudocode 1, and some adaptations are illustrated afterwards.

**Pseudocode 1:**
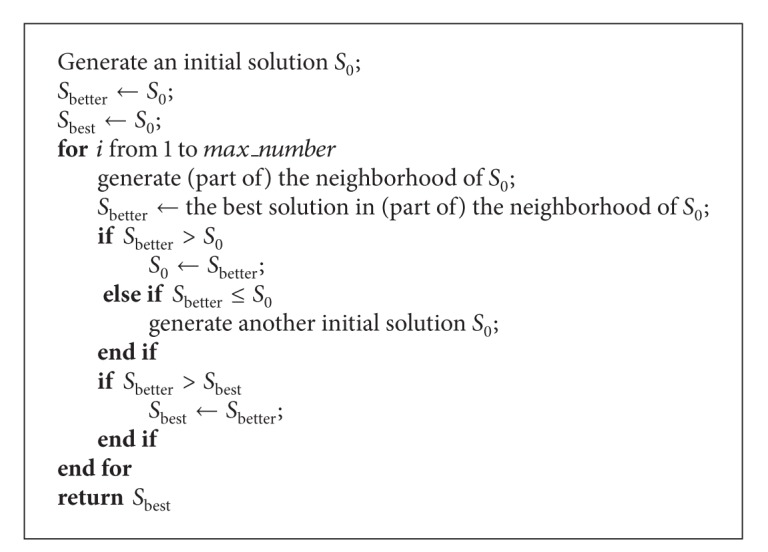
The pseudocode of the basic local search algorithm.

### 4.1. Generate an Initial Solution

As mentioned in many literatures [19–21], the initial solution has great effect on the performance of the local search algorithm. There are mainly two problems in generating the initial solutions: (a) how to generate a feasible solution; (b) how to generate a relatively good initial solution. For the first problem, we use the constraints in Definition 5 to make sure that all the initial solutions are feasible and belong to the desired-combination set *D*. For the second problem, we introduce an assumption that the combination of efficient DMUs is more efficient than the combination of inefficient DMUs. Therefore, when generating an initial solution, we choose an efficient DMU first and then add other DMUs to the combination randomly until the solution satisfies the constraints in Definition 5.

### 4.2. Generate a Neighboring Solution

The neighborhood of a solution may be too large, and usually only part of the neighborhood is generated during an iteration. As mentioned before, the vector *c*_*k*_ = [*c*_1*k*_, *c*_2*k*_,…,*c*_*nk*_]^T^ is used to denote the solution of CDMU_*k*_  (*k* = 1,2,…, |*D*|), and the DMUs set can be divided into two subsets according to their corresponding value in *c*_*k*_ as follows:

(14) $$\begin{array}{c} {{\text{CDMU}}_{k}}^{c}=\left\{{\text{DMU}}_{j}\;|\;c_{jk}=0,j=1,2,\ldots ,n\right\};\\ {\text{CDMU}}_{k}=\left\{{\text{DMU}}_{j}\;|\;c_{jk}=1,j=1,2,\ldots ,n\right\}. \end{array}$$

The way of generating a neighboring solution of CDMU_*k*_ in our adapted local search algorithm is to choose a DMU in CDMU_*k*_^*c*^ and a DMU in CDMU_*k*_ randomly and exchange their corresponding value in *c*_*k*_. The probability of choosing a DMU is determined by the individual efficiency of each DMU as follows:

(15) pj={Ej(∑DMUl∈CDMUkcEl)−1,   DMUj∈CDMUkc,(Ej−1)(∑DMUl∈CDMUk(El−1))−1,       DMUj∈CDMUk, j=1,2,…,n,

where *p*_*j*_ is the probability of choosing DMU_*j*_ and *E*_*j*_ is the individual efficiency of DMU_*j*_, in which *j* = 1,2,…, *n*. It should be noted here that the generated neighboring solution should also satisfy the constraints in Definition 5.

## 5. Numerical Examples

In this section, two numerical examples are provided to demonstrate the validity and effectiveness of our proposed model. By the first example, it is demonstrated that our proposed model is able to provide a unique best combination of DMUs and therefore would be more persuasive to the decision makers. By the second example, the superiority of our proposed model is demonstrated in different scenarios comparing with Cook and Green's model. What is more, the CEE problem considered as a 0-1 knapsack problem here is solved by the adapted local search algorithm in Section 4.

**Example 1 .**

The first numerical example has been introduced in Section 2.2 to illustrate the drawbacks of Cook and Green's model. It is used here again to demonstrate that, comparing with Cook and Green's model, our proposed model is able to provide a unique best solution to the decision makers and therefore would be more persuasive in practice. The data of four DMUs is provided once more in Table 4.

It is supposed that the input of CDMUs should be no more than 2 units and a comparison between Cook and Green's model and our proposed model is provided in Table 5.

The inefficient CDMUs are not shown in Table 5, and, by the application of our proposed model, the best combination we found is {1,2} which achieves a super efficiency of 1.2857. And by the comparison in Table 5, the validity and effectiveness of our proposed model can be demonstrated in three points: (a) some impractical CDMUs have been eliminated by our proposed model based on the fact that each DMU is allowed to appear only one time in a CDMU; (b) our proposed model is more powerful in discriminating the CDMUs and is able to provide a unique best combination which would be more persuasive to the decision makers; (c) in our proposed model, efficient CDMUs are prevented from being incorrectly evaluated by Cook and Green's model, such as the CDMU {3,4}.

**Example 2 .**

The second numerical example is selected from Oral's paper [6] in which 37 research and development projects in Turkish iron and steel industry were evaluated and selected collectively. This example was also used in Cook and Green's paper [5]. In this example, 37 projects are considered as the DMUs with one input and five outputs as follows:

 Input: the investment of a project;
 Output 1 (O1): direct economic contribution;
 Output 2 (O2): indirect economic contribution;
 Output 3 (O3): technological contribution;
 Output 4 (O4): scientific contribution;
 Output 5 (O5): social contribution.

The input and outputs data is provided in Table 6. It is supposed that the budget restriction is 1000 units and, as mentioned in [5], the average cost of these 37 projects is 67.99 and there would be approximately 15 projects selected in a combination.

By a searching process of our adapted local search algorithm, the best combination we found is {1,10,11,12,16,17,18,21,23,26,27,29,30,31,35,36}, and comparing with Cook and Green's result, our proposed solution achieves a better combinatorial efficiency of 1.3186 evaluated by our proposed model (13). A detailed comparison between Oral's solution [6], Cook and Green's solution [5], and our proposed solution is provided in Table 7.

The common projects selected by all three methodologies are defined as core projects according to Cook and Green's research [5], and in this numerical example, the core projects are {1,16,17,18,23,26,27,31,35,36}. In the meanwhile, the six distinct projects in our proposed combination are {10,11,12,21,29,30}. By comparing the costs of these three solutions, we can find that our proposed solution is better at making full use of the budgets. And finally, what is the most important, our proposed solution achieves the best combinatorial efficiency of 1.3186, and the combinatorial efficiency of Cook and Green's solution evaluated by model (13) is 1.2719 while Oral's solution is 1.2542.

**Figure 2:**
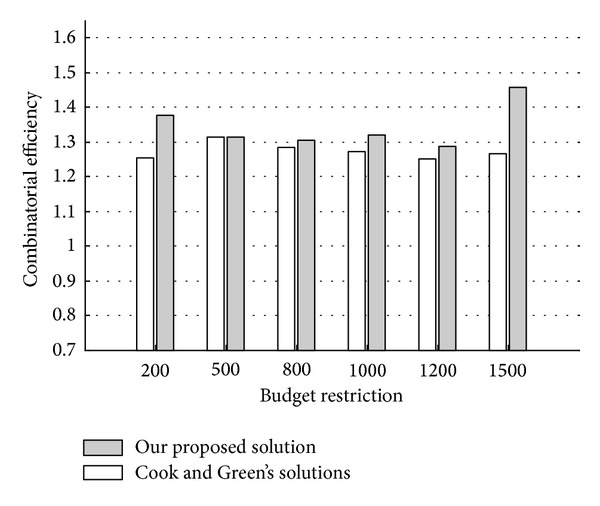
A comparison with different budget restrictions.

Some further comparisons between Cook and Green's model and our proposed model are provided in Table 8 (a~f) in which different budget restrictions are introduced into Example 2. The comparison with different budget restrictions is also shown in Figure 2.

By the comparison between Cook and Green's solutions and our proposed solutions with different budget restrictions; it can be found that our proposed model generally achieves a better combinatorial efficiency than Cook and Green's model and only when the budget restriction is 500 these two models achieve the same result. It should also be noted that the combinatorial efficiency scores calculated by a certain CEE model are incomparable according to different budget restrictions. For example, the combinatorial efficiency under budget restriction 200 is incomparable with the combinatorial efficiency under budget restriction 500, even for the same CEE model.

## 6. Conclusions

Data envelopment analysis (DEA) is generally an effective methodology of evaluating the relative efficiency of single decision making unit (DMU). However, in some practical problems, the decision makers are required to choose a group of DMUs instead of a single one. Therefore it is necessary to study the efficiency evaluation of multiple DMUs within a larger DMU set, and this relatively new problem is named as combinatorial efficiency evaluation (CEE) in our paper. By modifying some drawbacks in Cook and Green's model, a new combinatorial efficiency evaluation model is proposed based on the concept of knapsack problem and super efficiency model. Our proposed model is more logical in practice, and in the meanwhile, our proposed model is able to provide a unique best combination of DMUs which is more persuasive to the decision makers. Numerical examples are provided to demonstrate the validity of our proposed model compared with some other methods. It should be noted that our research in this paper is based on the CCR model and super efficiency model in constant return to scale (CRS) case, and some further studies are possible to extend the CEE problem into variable return to scale (VRS) case.

## Figures and Tables

**Table 1:** The data of four DMUs.

DMU	Input	Output 1	Output 2	Efficiency
1	1	1	5	1
2	1	3	4	1
3	1	5	2	1
4	1	4	1	0.8

**Table 2:** Different combinations of DMUs.

Combination set	CDMUs
*P*	{1}, {2}, {3}, {4}, {1,2}, {1,3}, {1,4}, {2,3}, {2,4}, {3,4}, {1,2, 3}, {1,2, 4}, {1,3, 4}, {2,3, 4}, {1,2, 3,4}
*R*	{1}, {2}, {3}, {4}, {1,2}, {1,3}, {1,4}, {2,3}, {2,4}, {3,4}
*D*	{1,2}, {1,3}, {1,4}, {2,3}, {2,4}, {3,4}
Model (10)	{1,1}, {2,2}, {3,3}, {4,4}, {1,2}, {1,3}, {1,4}, {2,3}, {2,4}, {3,4}

**Table 3:** Combinatorial efficiency evaluation by models (8) and (10).

CDMUs	Input	Output 1	Output 2	Combinatorial efficiency	
				Model (8)	Model (10)
{1,2}	2	4	9	1	1
{1,3}	2	6	7	0.9583	0.9285
{1,4}	2	5	6	0.8125	0.7857
{2,3}	2	8	6	1	1
{2,4}	2	7	5	0.8667	0.8571
{3,4}	2	9	3	1	0.9000

**Table 4:** The data of Example 1.

DMU	Input	Output 1	Output 2	Efficiency
1	1	1	5	1
2	1	3	4	1
3	1	5	2	1
4	1	4	1	0.8

**Table 5:** Combinatorial efficiency evaluation of Example 1.

CDMU	Input	Output 1	Output 2	Combinatorial efficiency	
				Cook and Green's model	Our proposed model
{1,1}	2	2	10	1	Impractical
{2,2}	2	6	8	1	Impractical
{3,3}	2	10	4	1	Impractical
{1,2}	2	4	9	1	1.2857
{2,3}	2	8	6	1	1.1111
{3,4}	2	9	3	0.9000	1.1250

**Table 6:** The data of Example 2.

Project	Input	O1	O2	O3	O4	O5	Efficiency
1	84.20	67.53	70.82	62.64	44.91	46.28	0.6543
2	90.00	58.94	62.86	57.47	42.84	45.64	0.5512
3	50.20	22.27	19.68	6.73	10.99	5.92	0.3360
4	67.50	47.32	47.05	21.75	20.82	19.64	0.5283
5	75.40	48.96	48.48	34.90	32.73	26.21	0.5064
6	90.00	58.88	77.16	35.42	29.11	26.08	0.6148
7	87.40	50.10	58.20	36.12	32.46	18.90	0.5060
8	88.80	47.46	49.54	46.89	24.54	36.35	0.4204
9	95.90	55.26	61.09	38.93	47.71	29.47	0.5177
10	77.50	52.40	55.09	53.45	19.52	46.57	0.5431
11	76.50	55.13	55.54	55.13	23.36	46.31	0.5618
12	47.50	32.09	34.04	33.57	10.60	29.36	0.5525
13	58.50	27.49	39.00	34.51	21.25	25.74	0.5045
14	95.00	77.17	83.35	60.01	41.37	51.91	0.6539
15	83.80	72.00	68.32	25.84	36.64	25.84	0.6518
16	35.40	39.74	34.54	38.01	15.79	33.06	0.8542
**17**	**32.10**	**38.50**	**28.65**	**51.18**	**59.59**	**48.82**	**1.0000**
18	46.70	41.23	47.18	40.01	10.18	38.86	0.7618
19	78.60	53.02	51.34	42.48	17.42	46.30	0.5179
20	54.10	19.91	18.98	25.49	8.66	27.04	0.3523
21	74.40	50.96	53.56	55.47	30.23	54.72	0.6022
22	82.10	53.36	46.47	49.72	36.53	50.44	0.5068
23	75.60	61.60	66.59	64.54	39.10	51.12	0.6754
24	92.30	52.56	55.11	57.58	39.69	56.49	0.5003
25	68.50	31.22	29.84	33.08	13.27	36.75	0.4024
26	69.30	54.64	58.05	60.03	31.16	46.71	0.6633
27	57.10	50.40	53.58	53.06	26.68	48.85	0.7420
28	80.00	30.76	32.45	36.63	25.45	34.79	0.3478
29	72.00	48.97	54.97	51.52	23.02	45.75	0.5784
30	82.90	59.68	63.78	54.80	15.94	44.04	0.5505
31	44.60	48.28	55.58	53.30	7.61	36.74	0.9459
32	54.50	39.78	51.69	35.10	5.30	29.57	0.6393
33	52.70	24.93	29.72	28.72	8.38	23.45	0.4299
34	28.00	22.32	33.12	18.94	4.03	9.58	0.7973
**35**	**36.00**	**48.83**	**53.41**	**40.82**	**10.45**	**33.72**	**1.0000**
36	64.10	61.45	70.22	58.26	19.53	49.33	0.7708
37	66.40	57.78	72.10	43.83	16.14	31.32	0.7391

**Table 7:** Combinatorial efficiency evaluation of Example 2.

Methodology	Solution		Budget (1000)		Combinatorial efficiency
	Common projects	Distinct projects	Consume	Remain	
Oral's	{1,16,17,18,23, 26,27,31,35,36}	{14,15,21,34,37}	964.70	35.30	1.2542
Cook and Green's		{6,14,15,32,34,37}	962.80	37.20	1.2719
Our proposed		{10,11,12,21,29,30}	975.90	24.10	1.3186

**Table:** (a) Budget restriction: 200

Methodology	Solution	Combinatorial efficiency
Cook and Green's	{16,17,31,34,35}	1.2544
Our proposed	{16,17,23,35}	1.3781

**Table:** (b) Budget restriction: 500

Methodology	Solution	Combinatorial efficiency
Cook and Green's	{16,17,18,23,26,27,31,34,35,36}	1.3132
Our proposed	{16,17,18,23,26,27,31,34,35,36}	1.3132

**Table:** (c) Budget restriction: 800

Methodology	Solution	Combinatorial efficiency
Cook and Green's	{1,14,16,17,18,23,26,27,31,32,34,35,36,37}	1.2837
Our proposed	{1,11,16,17,18,21,23,26,27,29,31,34,35,36}	1.3041

**Table:** (d) Budget restriction: 1000

Methodology	Solution	Combinatorial efficiency
Cook and Green's	{1,6, 14,15,16,17,18,23,26,27,31,32,34,35,36,37}	1.2719
Our proposed	{1,10,11,12,16,17,18,21,23,26,27,29,30,31,35,36}	1.3186

**Table:** (e) Budget restriction: 1200

Methodology	Solution	Combinatorial efficiency
Cook and Green's	{1,6, 12,14,15,16,17,18,21,23,26,27,29,31,32,34,35,36,37}	1.2504
Our proposed	{1,2, 11,13,14,16,17,18,21,23,26,27,29,31,32,34,35,36,37}	1.2880

**Table:** (f) Budget restriction: 1500

Methodology	Solution	Combinatorial efficiency
Cook and Green's	{1,2, 6,11,12,13,14,15,16,17,18,21,23,26,27,29,30,31,32,34,35,36,37}	1.2648
Our proposed	{1,2, 5,6, 7,9, 13,14,15,16,17,21,22,23,24,26,27,28,29,34,35}	1.4587
